# Research Progress and Development Direction of Filling Cementing Materials for Filling Mining in Iron Mines of China

**DOI:** 10.3390/gels8030192

**Published:** 2022-03-20

**Authors:** Hui Cao, Qian Gao, Xizhi Zhang, Bin Guo

**Affiliations:** 1 School of Civil and Resource Engineering, University of Science and Technology of Beijing, Beijing 100083, China; cao_h@ustb.edu.cn; 2 Hebei Iron & Steel Group Shahe Zhongguan Iron Ore Co., Ltd., Xingtai 054100, China; kyguobin@hbisco.com

**Keywords:** filling mining of iron ore, tailings filling, green filling cementitious material, research progress, development direction

## Abstract

Backfill mining is the only way to realize no-waste mining and create green mines, but complicated backfill mining technology, high mining costs, and low-production capacity greatly restrict its application in low-quality iron mines. To reduce the cost of iron ore backfill mining, a large number of low-cost green backfill cementing materials have been developed in China over the past 10 years. This paper first introduces the research and development of green cementitious materials using solid waste. Then, it points out the key technologies in the development of green filling cementing materials, reveals the hydration mechanism of green cementing materials through microscopic analysis and research, and optimizes the ratio of green cementing materials based on an orthogonal test. Finally, the development direction of green filling cementing materials is put forward: combining technology development with the filling mining method and filling process; taking the development route from technology to products and from products to commodities. To reduce the cost of filling mining and pursue the economic benefits of filling mining, a demonstration mine of tailings and green cementing materials is taken as the breakthrough point to comprehensively promote the development of iron ore full solid-waste filling mining technology and achieve its large-scale promotion and application.

## 1. Introduction

With China paying increasing attention to environmental protection and facing the development and utilization of more complex and difficult-to-mine mineral resources, not only is the number of nonferrous, gold, and precious metal mines using the filling mining method increasing year by year, but the filling mining method is also gradually expanding to iron ore and coal mines [1,2,3]. Especially over the past 10 years, China’s iron ore mountain filling mines have increased rapidly to promote the construction of green mines and waste-free mining [4,5,6,7,8,9,10]. Compared with the open-stope and caving mining methods, the filling mining method not only has complex mining technology and low-production capacity, but it also has high costs and poor economic benefits. With the development of beneficiation equipment and processes, the particle size of beneficiation tailings becomes increasingly finer, which not only increases the difficulty of slurry dehydration and the thickening cost but also increases the viscosity of tailings filling slurry and reduces the transportable concentration of pipelines, thus reducing the strength of tailings cemented filling bodies. To meet the requirements of safe mining production, the amount of cementitious material has to be increased, which leads to further increases in filling mining costs. Therefore, iron ore tailings filling mining faces more severe economic benefit problems.

The cement filling material is mixed with the underground filling material through the mixing and filling method. The filling slurry coagulates and solidifies in the stope to form a filling body with a certain strength to support the surrounding rock in the goaf, limit the deformation of the surrounding rock, and control the surface settlement to achieve the purpose of safe mining and disaster control. At the same time, the beneficiation tailings are backfilled into the underground goaf to avoid the environmental pollution caused by the stacking of tailings on the surface. At the same time, it also reduces the land acquisition, construction, and maintenance costs of the tailings pond and fundamentally solves the problem of geological disasters such as the breaking of a tailings dam and debris flow. Filling mining also reduces ore dilution and loss as well as realizing the full recovery and low-cost utilization of valuable resources. It can be seen that iron ore filling mining can “control harm with waste” and achieve the dual purposes of safe production and environmental protection.

At present, cement cementitious materials are still widely used in iron ore filling mining in China. Due to the poor adaptability of cement to the aggregate containing mud tailings, the strength of cemented backfill is low. The finer the tailings, the lower the strength of cemented backfill. At the same time, the viscosity of the tailing filling slurry increases with the smaller particle size of the tailing sand, increasing the pipeline transportation resistance of tailing filling slurry, decreasing the pipeline transportation concentration, and further reducing the strength of the cemented filling body. Therefore, the amount of cementitious material must be increased to further improve the cost of filling and mining.

To solve the problem of the poor economic benefits faced by cement tailings cemented filling mining, most filling mines mostly use graded tailings filling, and the particle size of filling tailings is increased by removing fine mud tailings from tailings. However, graded tailings filling not only reduces the utilization rate of tailings (generally 50%) but also causes it to be difficult for the graded discharged fine mud tailings to be stacked on the surface, which has the potential of more severe geological disaster risk. It can be seen that the research and exploration of low-cost filling cementitious materials suitable for tailings filling aggregates are not only necessary but also imminent for iron ore mountain filling mining. In the past 10 years, some progress has been made in the development of low-cost green filling cementitious materials with metallurgical slag. Among them, new filling cementitious materials—such as cementation powders—have been industrialized in some mines in China. This paper summarizes the research and development of filling cementitious materials and their application in iron ore, puts forward the key technologies for the development and utilization of new filling cementitious materials, and finally points out the development direction of the application of green filling cementitious materials in tailings filling mining.

## 2. Research in and Progress of Green Filling Cementitious Materials

Cement used for tailings filling cementitious material has the advantages of low strength, large dosage, high mining cost, and poor economic benefit. Therefore, using metallurgical solid waste resources to develop low-cost cement substitutes is an important research topic of filling mining.

### 2.1. Development and Utilization of Filling Cementitious Materials

#### 2.1.1. The Cementitious Material of Cement–Slag

Blast furnace slag is a type of solid-waste slag discharged from a blast furnace utilized for smelting pig iron [11]. After the discharge of blast furnace slag, it is usually water quenched; thus, it is also called water-quenched slag. According to the classification of its chemical composition, blast furnace slag belongs to silicate material, and its structure is a polymer of the crystalline phase and glass phase. The glass phase is the active component, while the crystalline phase is the inert component; therefore, the more content of the glass phase in the slag, the higher its activity. The main active components of blast furnace slag are calcium oxide, magnesium oxide, silicon oxide, and alumina. When the blast furnace slag is ground to the fineness of cement or above, it has potentially high water-hardening activity. Lime, cement, calcium hydroxide, or gypsum are used for excitation and activation to produce hydraulic cementitious materials [12]. Based on this, blast furnace slag can be used as a filling cementitious material. As early as 1994, the Zhangmatun iron mine of the Jinan Iron and Steel Company first carried out a strength test of a cemented backfill of mixed cementitious material with slag powder replacing part of the cement. The test results showed that based on the tailings filling aggregate when the cement–sand ratio was 1:7, the strength of a cemented backfill of the slag–cement-mixed cementitious material decreased in the early stage and increased in the later stage with an increase in the amount of slag replacing cement [13].

#### 2.1.2. The Cementitious Material of Cement–FA

Fly ash is the fine ash recovered from the flue gas in coal combustion, also known as fly ash. It is the solid waste discharged from coal-fired power plants. Its mineral composition is similar to that of high-alumina clay. Most of the fly ash is glass phase with a small amount of unburned carbon and some crystalline minerals of quartz and mullite. The ratio of calcium oxide (CaO) to silicon dioxide (SiO_2_) is approximately 0.1. The activity of fly ash depends on its fineness. The finer the fly ash, the higher its activity [14]. The research shows that under the action of alkali excitation, fly ash not only acts as the “micro aggregate” of cement but also has the cementation of “low-grade cement”, which optimizes the microstructure and mechanical properties of cemented backfill. The test results of fly ash–cement-mixed cementitious material show that fly ash can not only enhance the later strength of cemented backfill but also significantly improve the pumping performance of filling slurry to significantly reduce the pipeline transportation resistance of filling slurry [15,16,17,18,19]. Fu Yi et al. [20] introduced a proportion and production method of high-content fly ash cement. Hu Jiaguo et al. [21] studied the effect of activators on fly ash–cement cementitious material. The results show that when the cement, fly ash, and tailings ratio is 1:2:6, 1:2:8, and 1:2:10, respectively, and the composite activator of 0.3% lime + 2% gypsum + 0.5% CaCl_2_ is added, the 7 and 28 d strengths of the cemented filling block increase by approximately 45%, respectively, and the later strength is also increased by 17–32%. Through experimental research, Gou Mifeng et al. [22] determined that the best ratio of fly ash roadway side filling cementitious material is a sulfoaluminate cement content of 40%, a gypsum content of 20%, a lime content of 6%, and a fly ash content of 34%. It can be seen that the preparation of mixed cementitious material with fly ash as cement admixture can replace part of the cement and reduce the cost of filling cementitious material. However, the overall consumption is less, which is not very significant in improving the economic and environmental benefits of filling mining.

### 2.2. Research on High-Water Filling Cementitious Material

High-water quick-setting material has the properties of quick setting and early strength and can produce hydraulic hardness under the condition of a high water–cement ratio. Water, at nine times the high-water material’s volume, can be condensed into a solid to form a highly water-hydrated calcium sulfoaluminate product with a certain strength [23,24]. In the 1960s, British scholars successfully developed it for the first time and used it for coal mine roadway filling support. In the 1990s, Sun Henghu of the China University of Mining and Technology developed high-water consolidated filling material with a water–cement ratio of 3.0 (referred to as high-water material) and successfully carried out an industrial filling test in the Zhaoyuan gold mine, Shandong Province [25]. The test results showed that the concentration range of filling slurry consolidated by high-water materials is from 30% to 70%. The filling slurry can condense into solidly cemented backfill without dehydration in the filling stope and has the characteristics of fast solidification and high early strength. However, the source of high-water materials has great limitations and a high filling cost, and the cemented filling body can easily be weathered and decomposed in the atmospheric environment, making them difficult to be popularized and applied in the filling mining of metal mines [26].

### 2.3. Development and Application of Green Filling Cementitious Materials

Compared with cement, green filling cementitious material is a new type of filling cementitious material prepared by using alkali, salt, or composite activators to stimulate a class of potentially active pozzolanic solid wastes, such as slag, fly ash, coal gangue, steel slag, and red mud, to produce hydration reactions [26]. The research on new cementitious materials can be traced back to the “soil cement” proposed by Glukhovsky of the former Soviet Union, in addition to the cementitious material called “geopolymer” by Davidovits of France [27]. In recent decades, a variety of green filling cementitious materials have been developed using pozzolanic solid wastes, such as slag, steel slag, fly ash, calcined coal gangue, and red mud, which significantly reduces the cost of filling cementitious materials and has innovated the tailings filling technology as well as production process to a certain extent [28,29,30].

#### 2.3.1. Slag-Based Filling Cementitious Material

An activator is used to stimulate the potential activity of blast furnace slag to produce a hydration reaction and produce ettringite with the cementitious property. The cementitious material prepared from this is called slag-based cementitious material. The Changsha Institute of Mining Research Co., Ltd., Changsha, China, used lime and other activators to research filling cementing agents, which greatly improved the strength of tailings cemented filling bodies. The results showed that the 28 d strength of tailings cement with a cement–sand ratio of 1:8 can reach more than 2 MPa, and its cost is only 40–60% of the cost of cement. The Jiaojia gold mine in Shandong Province carried out experimental research on slag-based cementitious material and developed cementitious powder green filling cementitious material in 2003. The test results showed that the cementitious powder cementitious material meets the technical requirements of tailings filling, and its material performance and price are better than that of cement. It has been popularized and applied in Shandong Jiaojia, Xincheng, and other gold-filling mines [31]. Gao Qian and others prepared composite activators with lime and desulfurized gypsum, respectively, for full tailings and coarse aggregates, carried out strength tests of the cementitious body of slag-based cementitious materials with different ratios, and developed consolidated powder cementitious materials [32]. The hydration products and internal structure of cement at different ages were analyzed. The results showed that [33,34] the alkaline slurry formed by the lime alkali activator creates conditions for the dispersion and dissolution of slag glass. Under the hydrolysis of lime, a large number of Ca^2+^ and OH^−^ ions are produced. Ca^2+^ and –OH in the slurry enter the water film on the slag surface to form a layer of an alkaline membrane solution. At this time, the concentration of OH^−^ ions is very large, which are more capable than water molecules of entering the internal holes of the slag–glass network structure to promote the dispersion, dissolution, and hydration of slag. Firstly, the hydration process of slag is the hydration reaction between the SiO_2_ and Al_2_O_3_ active mineral components in slag and Ca(OH)_2_. The water in the mixture is continuously supplied, and an alkaline film is continuously formed on the surface of the slag; through the gaps between the hydration products on the slag surface, it penetrates inward to corrode the slag until all the slag is hydrated. Gypsum can stimulate the salt of slag-based cementitious materials and promote the reaction between Al_2_O_3_ and Ca(OH)_2_ in slag to produce calcium aluminate hydrate, namely, ettringite. Since the formation of ettringite consumes the main hydration product, calcium aluminate, after slag hydration, the slag hydration process is accelerated. At the same time, the newly formed hydrated calcium sulfoaluminate increases the compactness of the structure. The product has a high water content, which greatly reduces the free water and causes the structure to become increasingly compact to improve the strength of the cemented backfill.

#### 2.3.2. Fly-Ash-Based Filling Cementitious Material

Fly-ash-based filling cementitious material is one of the research contents of filling mining technology [35]. The Xinqiao pyrite mine has previously used fly ash for mine filling [36,37]. Zhang Lei and Chen Xianshu have, respectively, carried out research on fly-ash-based cementitious materials and developed cementitious materials with different ratios [38,39]. The ratios and strength effects are shown in Table 1.

Cementitious materials were prepared from fly ash, silicate gel C–S–H seeds, composite sulphates, and sulfoaluminates and admixtures. The cementitious materials were mixed according to a 40–80% fly ash, 10–30% silicate gel C–S–H seed, 5–15% composite sulfate and sulfoaluminate, and 1–5% additive mixture. When the content of a +45 μm powder is zero, a fly-ash-based gelling agent can be prepared to replace cement. Feng Juen et al. used cement, gypsum, lime, admixture, and fly ash to research filling cementitious materials [40]. When the content of fly ash was 10%, the early strength of cement was the same as that without fly ash; however, when the content of fly ash reached 20%, the early strength of the cement decreased, but the later strength increased. When the content of fly ash reached 30%, its early strength decreased significantly, but the 90 d strength was equivalent to that without fly ash. Chen Weixin et al. conducted experimental research on belt filling mining with fly ash and cement [41]. The results showed that when the content of fly ash was 80–89%, an HJJ activator was used, and a small amount of sulfoaluminate cement, KYY-ZHZ early strength retarder, KYY-S accelerator, lime, gypsum, and other composite activators were added. Under the conditions of activation for 2 to 8 h and a liquid–solid ratio of 0.95:1.25, the 8 h compressive strength of the cemented backfill was more than 0.7 MPa, the 3D compressive strength was more than 2 MPa, and the 28 d compressive strength was more than 5 MPa.

In conclusion, fly ash in fly-ash-based cementitious materials has the dual effects of potential activity and a microaggregate effect. Low-strength filling cementitious materials can be prepared by optimizing the proportion and proper grinding. However, due to the low early strength, it has not been popularized and applied.

#### 2.3.3. Red-Mud-Based Filling Cementitious Material

Red mud is the waste residue discharged from the aluminum industry. Its main minerals include dicalcium silicate, tricalcium aluminate, calcium carbonate, hydrated calcium aluminosilicate, and hematite. Therefore, it has a certain potential activity, but its activity varies with the production methods of alumina, its origin, and the grade of bauxite [42]. Due to the large demand for cementitious materials in filling mines and the low strength requirements of cement, green filling cementitious materials can be developed by using red mud [43]. Shandong Aluminum Co., Ltd., cooperated with Changsha Institute of Mining Research Co., Ltd., to carry out research on red-mud-based cementitious material by mixing red mud, fly ash, and lime by sintering and carrying out filling tests in the Hutian aluminum mine. However, due to the low early strength of cemented backfill, it could not meet the filling requirements of the mines; therefore, it cannot be applied in mines [44]. Huang Di et al. carried out experimental research by using tailings and sintering red mud and discerned that the optimal ratio of red-mud-based cementitious materials is 49.2% red mud, 32.8% slag, 10% cement clinker, and 8% desulfurization gypsum. The results of differential scanning and thermogravimetric analysis showed that the hydrated products of ettringite and C–H–S gel produced in the early stage of hydration were beneficial for increasing the early strength of the cementation [45], and the strength of cemented backfill decreased sharply as the ratio of cement to sand decreased. Liu Ying used Bayer red mud to develop red-mud-based cementitious materials. The water-hardening characteristics of Bayer red mud in the calcium carbide slag gypsum system were studied through XRD, FTIR, SEM, and ICP-OES. The results showed that the composite activator prepared from calcium carbide slag and desulfurized gypsum can stimulate the potential activity of Bayer process red mud and produce a hydraulic cementation reaction. The 28 d strength of the cementite test block reached more than 7 MPa [46]. The test results of red-mud-based cementitious material carried out by Yu Haitao showed that red mud has good water retention performance, a large proportion range, long-term strength performance, good water retention of slurry, small pipeline wear, and can heal small cracks in the pipeline [47].

In conclusion, compared with slag- and fly-ash-based cementitious materials, red mud has low potential activity and high technical difficulty in development. For fine tailings filling aggregates, the strength of cemented filling bodies is very low; thus, red-mud-based filling cementitious material is still in the research stage.

## 3. Key Technologies for the Development of Green Filling Cementitious Materials

### 3.1. Research Background of Green Filling Cementitious Material

Usually, new filling cementitious materials use lime, cement clinker, and admixture to prepare an activator that can stimulate the potential activity of blast furnace slag and produce a hydration reaction to form a stone body. At present, slag-based cementitious materials, such as cementitious powder, have consolidation agents, and consolidation powders have been developed. In recent years, with the strict management of China’s environmental protection and the restrictions on the production and energy reduction of iron, steel, and cement enterprises, the output of blast furnace slag is decreasing, and its application in building materials is increasing. Not only is the utilization cost of blast furnace slag increasing year by year, but the demand is also in short supply in some areas. Therefore, with the increase in the cost of slag-based cementitious materials, it is close to ordinary Portland 42.5 cement. On the other hand, the metallurgical industry discharges a large number of low-quality solid wastes, such as steel slag, white slag, magnesium slag, and industrial by-product gypsum, every year. Due to the fact of its poor quality, low activity, and adverse mineral components, such as toxic and harmful substances, the utilization of low-quality solid wastes is difficult, has a high cost, and has a low utilization rate. In 2016, the output of steel slag in China was approximately 65–120 million tons, while the utilization rate was only approximately 20% and the storage volume of steel slag was as high as 1 billion tons. The main chemical components of steel slag are CaO, MgO, SiO_2_, Fe_2_O_3_, MnO, Al_2_O_3_, and a small amount of TiO_2_ and P_2_O_5_. The main mineral composition is tricalcium silicate, dicalcium silicate, calcium magnesium olivine, calcium merwinite, dicalcium ferrite, RO (oxide of magnesium, iron, and manganese), free lime (*f*—CaO), etc. Therefore, steel slag has certain potential activities. With the gradual increase in filling mines in China, the demand for filling cementitious materials is increasing year by year, and the demand for filling cementitious materials is increasing. According to the characteristics of filling cementitious materials, research on green filling cementitious materials using low-quality solid waste can not only significantly reduce filling mining costs and alleviate the shortage of high-quality slag resources but also explore a method for the modeling of low-quality solid waste and the utilization of high added value.

### 3.2. Research and Development of Green Filling Cementitious Materials

As the name suggests, green filling cementitious material is a low-cost green filling cementitious material developed entirely from solid waste. Alkaline or salt solid waste is used to develop activators instead of lime and clinker to stimulate the hydration reaction of potentially active solid waste to form a stone body. Therefore, green filling cementitious materials are more different than slag-based cementitious materials in excitation mechanisms, material ratios, and water-hardening reactions. To develop green filling cementitious materials with reliable technology and reasonable economy, technical developments and research are put forward.

#### 3.2.1. Technological Research and Development

Developing technology is an important step in the research of green filling cementitious material. It mainly uses alkali and salt solid wastes to develop activators as well as proportion testing and the optimization of cementitious materials:

(1) Firstly, the mineral composition and characteristics of the available solid waste are analyzed, and then the alkalinity and salinity of the solid waste are quantitatively classified and comprehensively evaluated. The same is the case for the pretreatment and safety evaluation of the available solid waste;

(2) Research on green filling cementitious material tests and proportions is conducted, and then strength tests on the cementitious block according to different proportions of green activators are conducted. On this basis, the proportion of composite activators is optimized; finally, the economy, environmental protection, and safety of cementitious materials are analyzed;

(3) According to different tailings filling materials, carry out the test of the cement strength and pipeline transportation characteristics of green filling cementitious materials, study the relationship between tailings fineness, cement–sand ratio, slurry concentration, cement strength, and slurry pipeline transportation characteristics, and evaluate the safety and reliability of green filling cementitious materials.

#### 3.2.2. Production Process

The production process is a key step in the transformation of green filling cementitious materials from technology to products. The grinding process of cementitious materials and product quality analysis as well as evaluation is mainly designed as follows:

(1) The production technology of green filling cementitious material is studied. According to the physicochemical characteristics, hardness, and grindability of green filling cementitious materials, the appropriate grinding equipment, production process, and quality indices are selected. On this basis, considering comprehensive factors, such as equipment investment, production scale, and economic benefits, the investment, operation cost analysis, and decision making on green filling cementitious materials are carried out;

(2) The quality index of green filling cementitious material is studied. According to the grinding equipment and production process, the quality indices of green filling cementitious materials are determined including the physicochemical characteristics, quality and activity, powder fineness, and ratio control accuracy of raw materials.

#### 3.2.3. Product Commercialization

The commercialization of products is based on the industrialized production of green filling cementitious materials. This is to popularize and apply the products as commodities in filling mines. It mainly involves products’ technical indicators, application conditions, and detection methods:

(1) Technical indices of green filling cementitious materials including the particle size parameters and particle size gradation, powder density, and fluidity of cementitious materials;

(2) Quality index of green filling cementitious materials including product storage conditions, shelf life, comparative analysis with cement in terms of technology and economy, and precautions for product application.

### 3.3. Research Contents of Green Filling Cementitious Materials

#### 3.3.1. Characteristic Analysis and Treatment of Solid Waste

Green filling cementitious material is prepared by using solid wastes, such as alkali and sulfate, to prepare composite activators and stimulate the activity of slag powder. The activity of raw materials is related to the performance of green filling cementitious materials. Therefore, mineral composition analysis, acid–base classification, and the toxic as well as harmful treatment of the used solid waste is one of the important research contents of green filling cementitious materials.

#### 3.3.2. Proportion Optimization Test of Green Filling Cementitious Material

The key to the development of green filling cementitious materials lies in the optimization and decision making of the activator ratio. The physicochemical properties and utilization cost of cementitious materials are closely related to their grinding fineness and production technology; therefore, it is necessary to carry out a strength test of cemented backfill according to different fineness and ratios as well as optimize the ratio of green filling cementitious material on this basis.

#### 3.3.3. Grinding Process of Green Filling Cementitious Material

The application of green filling cementitious material as a product in filling mining depends on the material production process. To this end, the following research contents are involved:

(1) Composite activator grinding and mixing homogenization process. Generally, activator materials are different in hardness, moisture content, particle size, and particle size gradation; therefore, it is necessary to study the grinding and preparation processes of composite activators;

(2) Mixing the homogenization processes of the activator and active material. For green filling cementitious materials produced by different grinding processes, a mixing system needs to be used to mix and homogenize different powder materials to produce green filling cementitious materials. Therefore, mixing equipment and production processes affect the homogenization effect and quality of green filling cementitious materials.

## 4. Research and Development Direction of Green Filling Cementitious Materials

### 4.1. Problems Faced by Research on Green Filling Cementitious Materials

With the wide application of filling mining and facing the problems of a shortage of slag resources in addition to high utilization costs, the development of green filling cementitious material using low-quality solid waste is another research hotspot of filling mining in recent years. Du Huihui et al. [48] used vanadium titanium slag, steel slag, and desulfurization gypsum of the Chengsteel Company to carry out the strength test of filling test blocks with different slag contents and curing temperatures and obtained the optimized formula of green filling cementitious material: vanadium titanium slag, 58%; steel slag, 30%; desulfurization gypsum, 12%. Cui Xiaowei et al. [49] further studied the hydration reaction mechanism of vanadium titanium steel slag-based green filling cementitious material. The results showed that the pH value of the hydration solution of the green filling cementitious material decreased first and then increased with an increase in the reaction age. The early concentration of Ca^2−^ and silicon (aluminum) solute was low, and the later concentration increased. Under the combined excitation of desulphurized gypsum, slag and slag promoted hydration. The hydration products were mainly ettringite (AFt) and calcium silicate hydrate (C–S–H) gel, and the number of hydration products increased rapidly. The needle-like AFt crystals interspersed in C–S–H gel caused the hardened paste structure to become more compact. Li Litao et al. [50] researched steel-slag-based green filling cementitious material by using steel slag, slag fly ash, and desulfurization gypsum for a fine aggregate of iron ore tailings. By establishing the proportion optimization model of green filling cementitious material and using a genetic algorithm for global optimization, the optimal proportion of green filling cementitious material was obtained as follows: desulfurization gypsum, 20%; steel slag, 33%; fly ash, 25%; slag, 22%. The 7 and 28 d strengths of the tailing backfill reached 1.38 and 3.56, respectively. The relationship between the loss rate of C–S–H gel and ettringite in a green filling cementitious material system was studied. The results showed that the strength of the filling body increased when the loss rate of ettringite increased from 3.64% to 8.7%. Liang Feng et al. [51] carried out experimental research on steel-slag-based consolidated powder cementitious material by using steel slag, slag, and desulfurization gypsum for a fine-tailings aggregate of the Sishanling iron mine. An orthogonal design was used to test the strength and volume shrinkage of the cementitious body. The optimized formula of consolidated powder cementitious material was obtained as follows: steel slag, 40%; gypsum, 22%; slag, 38%. The 28 d strength of the tailings cemented backfill was more than 1.5 MPa. On the contrary, the strength of the gypsum filling was greater than that of the gypsum filling for 287 d; the volume shrinkage rate of the filling body was less than 6% in 28 d, but there was a slight expansion in the later stage.

### 4.2. Development Direction of Green Filling Cementitious Materials

Green filling cementitious material is still in the stage of research and exploration. With the support of a special fund for major technology transformation from the Department of Science and Technology of Hebei Province, the researchers in this paper developed steel-slag-based consolidated powder cementitious material, which was successful in the industrial filling test of the Zhongguan iron mine and realized its industrial application. To popularize the application of green filling cementitious materials in filling mines, further research and development directions are put forward.

#### 4.2.1. Development and Research of Multi-Solid Waste Green Filling Cementitious Material

At present, green filling cementitious material uses steel slag and desulfurized gypsum to prepare a composite activator to stimulate the activity of slag powder and develop steel-slag-based green filling cementitious material. As is known, industrial solid wastes include white slag, nickel slag, magnesium slag, calcium carbide slag, and other alkaline solid wastes, in addition to steel slag and desulfurization gypsum. At the same time, the chemical industry also discharges phosphogypsum, fluorine gypsum, and other salt wastes. The characteristics of industrial solid waste are not only scattered sources, large output, and complex composition, but some solid wastes also have the characteristics of toxicity, radioactivity, and corrosivity. According to the development experience of steel-slag-based consolidated powder cementitious materials, the development of green filling cementitious materials by using a variety of alkali and salt solid wastes is the research and development direction of green filling cementitious materials. Liu Quan and Huang Xuquan researched fluorine–gypsum-based cementitious materials [52,53]. Li Gaolu and Ni Wen et al. [54,55] researched the influence law of calcium carbide slag on filling cementitious materials and fluorogypsum-based green filling cementitious materials. In this paper, the author carried out an exploratory study on green filling cementitious material using refined white slag. The results showed that white slag alkaline solid waste has the same alkali excitation effect as steel slag as well as the fact that white-slag-based green filling cementitious material can be developed. The results showed that magnesium slag can also be used as an alkali activator to develop green filling cementitious material.

#### 4.2.2. Study on the Hydration Mechanism of Green Filling Cementitious Material

The core technology of the development of green filling cementitious materials is to develop composite activators by using alkali and salt solid wastes to stimulate potential activity and prepare green filling cementitious materials. Obviously, due to the different mineral compositions and salinity of different solid wastes, the hydration mechanisms of activators are different. Therefore, research on the hydration mechanisms of a variety of green filling cementitious materials is the basis of optimizing the proportion of cementitious materials and the research direction of developing green filling cementitious materials. Ni Wen et al. [48] studied the hydration mechanism of green filling cementitious material with slag electric furnace reduction slag. Li Litao et al. [51] carried out intelligent decision-making research on the proportion optimization of green filling cementitious materials.

#### 4.2.3. Research on Green Filling Cementitious Materials in Combination with Filling Mines

Cementitious materials suitable for mining methods and the production conditions of filling mines are the basis for industrial application in mines. As is well established, different mining materials have different methods, technical conditions, and mining processes, and there are great differences in the requirements for the characteristics of cementitious materials. Therefore, the development of green filling cementitious materials must be closely combined with mines; according to the filling aggregate, strength requirements of filling bodies, slurry pipeline transportation conditions, preparation methods, and transportation technology, it is the only way to carry out the research and development of green filling cementitious materials. At the same time, the characteristics of green filling cementitious material research are to use a variety of solid wastes according to local conditions and carry out the development and application of research on new cementitious materials.

#### 4.2.4. Along the Development Route of Technology, Products, and Commodities

The industrial application of green filling cementitious materials lies not only in transforming technology into products but also in transforming products into commodities. From technology to products, and from products to commodities, is an important development direction of green filling cementitious materials. The research includes the following: according to the optimized formula of green filling cementitious material, combined with the mining method and production technology of mine filling, carry out industrial tests on the strength and pipeline transportation characteristics of the cemented filling body, test and verify the reliability, feasibility, economy, and environmental protection of filling cementitious material, and finally obtain approval for filling mines as a green filling cementitious material recognized in the market, thus achieving circulation in the market.

#### 4.2.5. Create a Green Filling Mining Demonstration Mine

As a low-cost filling cementitious material, it is necessary to establish a demonstration project for its popularization and application in filling mines. It not only provides filling mines that can visit and exchange technology for tailings filling but also plays an exemplary role in the development and popularization of green filling cementitious materials. The contents involved in the construction of the demonstration project: select typical tailings filling mines and carry out the development of green filling cementitious materials and industrial filling tests according to the mining methods, mining technical conditions, filling aggregate, filling system, filling multiple lines, and the requirements of the filling stope for filling body strength and slurry pipeline transportation. Through a large-scale industrial filling test, the corresponding tailings filling cementitious material is developed, and the filling process, technical specifications, and quality standards of new cementitious materials are formulated. On this basis, the technical specification and safety production plan for tailings filling are prepared to lay a foundation for the popularization and application of low-cost green filling cementitious materials in mines.

## Figures and Tables

**Table 1:** Ratios and strength effects of common fly-ash-based cementitious materials.

Number	Clinker (%)	Fly Ash (%)	Slag (%)	Other (%)	Strength Effect
1	18	10	72	0	≥P.O 42.5
2	14	30	56	3	
3	18–20	40–45	30–36	6 to 7	≥P.O 32.5

## Data Availability

All data, models, and code generated or used during the study appear in the submitted article.
